# Using mobile phone text messaging for malaria surveillance in rural Kenya

**DOI:** 10.1186/1475-2875-13-107

**Published:** 2014-03-19

**Authors:** Sophie Githinji, Samwel Kigen, Dorothy Memusi, Andrew Nyandigisi, Andrew Wamari, Alex Muturi, George Jagoe, René Ziegler, Robert W Snow, Dejan Zurovac

**Affiliations:** 1 Malaria Public Health Department, KEMRI-Wellcome Trust - University of Oxford Collaborative Programme, Nairobi, Kenya; 2 Division of Malaria Control, Ministry of Health, Nairobi, Kenya; 3 Management Sciences for Health, Nairobi, Kenya; 4 Medicines for Malaria Venture, Geneva, Switzerland; 5 SMS for Life, Novartis AG, Basel, Switzerland; 6 Centre for Tropical Medicine, Nuffield Department of Clinical Medicine, University of Oxford, Oxford, UK; 7 Center for Global Health and Development, Boston University School of Public Health, Boston, MA, USA

**Keywords:** Malaria, Surveillance, Text messaging

## Abstract

**Background:**

Effective surveillance systems are required to track malaria testing and treatment practices. A 26-week study “*SMS for Life”* was piloted in five rural districts of Kenya to examine whether SMS reported surveillance data could ensure real-time visibility of accurate data and their use by district managers to impact on malaria case-management.

**Methods:**

Health workers from 87 public health facilities used their personal mobile phones to send a weekly structured SMS text message reporting the counts of four basic surveillance data elements to a web-based system accessed by district managers. Longitudinal monitoring of SMS reported data through the web-based system and two rounds of cross-sectional health facility surveys were done to validate accuracy of data.

**Results:**

Mean response rates were 96% with 87% of facilities reporting on time. Fifty-eight per cent of surveillance data parameters were accurately reported. Overall mean testing rates were 37% with minor weekly variations ranging from 32 to 45%. Overall test positivity rate was 24% (weekly range: 17-37%). Ratio of anti-malarial treatments to test positive cases was 1.7:1 (weekly range: 1.3:1–2.2:1). District specific trends showed fluctuating patterns in testing rates without notable improvement over time but the ratio of anti-malarial treatments to test positive cases improved over short periods of time in three out of five districts.

**Conclusions:**

The study demonstrated the feasibility of using simple mobile phone text messages to transmit timely surveillance data from peripheral health facilities to higher levels. However, accuracy of data reported was suboptimal. Future work should focus on improving quality of SMS reported surveillance data.

## Background

The WHO 2010 guidelines for treatment of malaria recommend universal parasitological diagnosis of all patients suspected of malaria and treatment of only those who test positive with recommended first-line therapy for uncomplicated malaria [1]. In 2012, the WHO Global Malaria Programme launched an initiative entitled “T3: Test-Treat-Track” which, in addition to testing and treatment, emphasized the need for the disease to be tracked using timely and accurate surveillance systems [2]. Unfortunately, surveillance systems are weakest in 41 countries where 85% of estimated malaria cases occur [3]. Current reporting and use of malaria surveillance information is delayed and imprecise [4] leading to typically low estimates of malaria incidence from routine surveillance data [5]. Effective surveillance systems to gather, store and process information from communities to national levels in a well-timed manner are urgently needed to monitor implementation of the new case-management policy [6-8]. With the rapid increase of mobile phone connections in developing countries, the potential of using mobile phone text messages for field data collection is now widely recognized for its immediate communication, ease of use and reducing data transmission delays [9-13].

In Kenya, routine malaria surveillance data are derived from various data reporting systems which include the District Health Information Systems (DHIS), Integrated Disease Surveillance and Response (IDSR), Logistics Management Information System (LMIS), and Laboratory Information Management System (LIMS) [14]. Data from these systems are reported at different intervals with wide variations across the systems. An analysis conducted by the Division of Malaria Control (DOMC) in 2011 recommended developing capacity to collect certain surveillance indicators directly from facilities as a short-term measure to improve data quality [15]. As one approach towards this realization, the DOMC piloted a project *SMS for Life*, using mobile phone text messages to transmit data directly from health facilities. The project followed a Tanzanian model where it was used to report on a weekly basis, stocks of artemether-lumefantrine (AL) and quinine [16]. In Kenya, the project was modified to include weekly reporting of stocks (AL and rapid diagnostic tests (RDT) and four basic malaria surveillance data elements (number of outpatients presenting at the health facility, number of outpatients tested for malaria, number of test positives and number treated with anti-malarials). To simplify reporting, the system was designed to receive stocks (AL and RDT) and surveillance data in separate text messages on different days. The experiences with the stock component of the project were published previously [17]. This paper reports on response rates, timeliness and accuracy of SMS reported surveillance data and describe trends in testing rates, test positivity rates and appropriate treatment ratios.

## Methods

### Study sites

The study was undertaken at all 87 public health facilities in five districts (Machakos, Msambweni, Manga, Ijara and Vihiga) over 26 weeks from August 2011 to March 2012. The districts were purposively selected by DOMC to represent different malaria endemicities, supply chain mechanisms and mobile phone network coverage. Msambweni and Vihiga represented high malaria endemic districts with seasonal variations and perennial transmission respectively. Machakos and Ijara represented low malaria transmission districts with seasonal peaks while Manga represented low, malaria epidemic area. Regarding the supply chain mechanisms, Machakos, Msambweni and Ijara districts operated a “pull” system where all health facilities made orders on a bimonthly (for hospitals) or quarterly basis (for health centres and dispensaries). Manga and Vihiga districts operated combination of a “push” system (quarterly supply of predetermined quantities for health centres and dispensaries) and “pull” system (bimonthly supply based on orders for hospitals). Machakos, Vihiga and Manga districts had universal mobile phone network coverage while Msambweni and Ijara had 92% and 38% of population covered with mobile network signal.

### Description of the *SMS for Life* system

**Figure 1:**
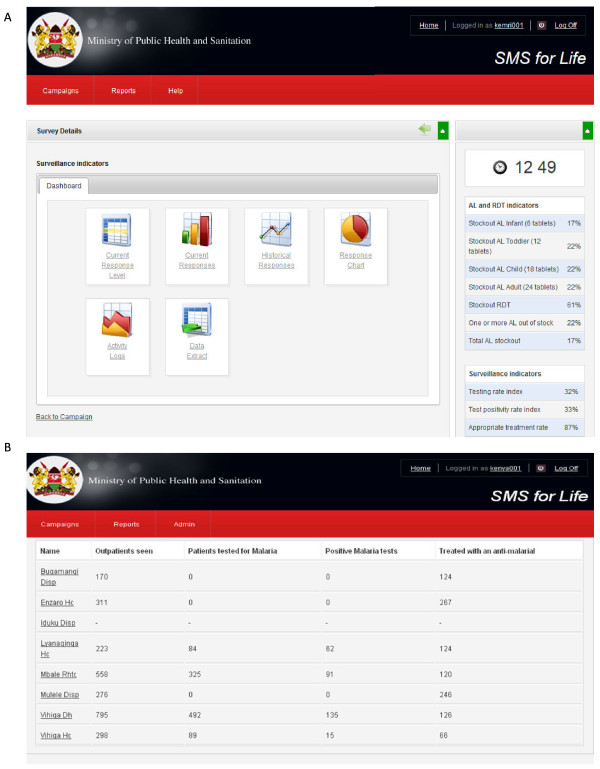
**Screenshots of *SMS for Life *** **web-based surveillance reporting tool (A) *SMS for Life *** **surveillance data dashboard; (B) Display of health facilities surveillance report for one week in one study district.**

The *SMS for Life* system is a web-based, real-time data reporting application composed of an SMS management tool and a web-based reporting application [16]. The SMS management tool stores the mobile phone number of one health worker from each registered health facility. It uses a free short code number that enables registered health workers to receive a weekly data request text message and send a standard format SMS reply with counts of data requested at no cost. The web-based reporting tool captures the data sent via SMS by the registered health workers and makes it available in real-time through the internet to designated members of the District Health Management Team (DHMT) and national officials at DOMC. The DHMT members directly access the password-protected *SMS for Life* website to monitor malaria surveillance parameters of each health facility within their district, while DOMC officials at national level monitor the same in all districts participating in the project. The website also provides current and historical data on reported surveillance parameters and summaries of testing rates, test positivity rates and appropriate treatment rates at each health facility and aggregated at the district level (Figure 1A). DOMC officials and DHMT members also receive a weekly email sent to their smart phones from the *SMS for Life* website summarizing all the data reported in the previous week. This enables them to monitor data anytime even in the absence of internet connectivity once the email has been received.

### Project implementation

#### ***Health worker training***

The training for health workers involved one day refresher course on malaria case management and one day training on *SMS for Life* data reporting procedures. An invitation letter was sent by DOMC through the DHMTs to in-charges of all public facilities in the study districts asking them to attend or nominate another health worker to participate is the *SMS for Life* training and to bring their personal mobile phones. DHMT members involved in malaria control (district malaria coordinator, district medical laboratory technologist or district clinical officer) were also invited for the training so that they could provide support to front line health workers during the project implementation. DHMT members received further half day training on how to access the web database to view surveillance data reports and indicators from individual facilities as well as aggregated data for the whole district. The case management training focused on algorithms recommending testing of all patients suspected of having malaria, treatment of test positive patients with recommended first-line anti-malarial and a practical session on how to perform parasitological diagnosis using RDT. *SMS for Life* data reporting training involved registration of the mobile phone numbers of participating health workers and their facilities into the *SMS for Life* system. The health workers were guided on how to extract surveillance data elements from three sets of mock registers developed to mimic outpatient, laboratory and RDT registers used at health facilities in Kenya. For each set of registers, health workers received a data request message upon which they counted the total number of outpatients recorded (OP), total number of outpatients prescribed any anti-malarial treatment (AM), total number of outpatients tested for malaria (TM), and total number of outpatients positive for malaria (PT). Each health worker composed a standard format SMS comprising the parameter code (OP, AM, TM, and AM) followed by the number of patients counted after each code and sent the text to the designated *SMS for Life* free code number. After each set of registers, both trainers and participants reviewed the counting process to establish the correct counts. Participants whose counts were incorrect were helped to identify their mistakes and rectify them in the subsequent mock register counts. At the end of the training, each participant received a reference job aid highlighting the key steps of counting and sending the *SMS for Life* text messages.

#### ***Data reporting procedures***

Following the training, registered health workers received a data request SMS text message every Monday at 14.00, counted for the preceding week (Monday to Sunday) the four surveillance data elements and sent a standard format text message comprising the data counts to the designated free code number. Health workers who did not reply to the data request message received an automatic reminder on Tuesday at 14.00. Those responding to the data request message within 27 hours from the time of the first request got an incentive of 50 Kenya shillings (0.6USD) worth of airtime if they also sent a similar stock message by Friday 17.00 in the same week [17]. Data were received into *SMS for Life* web database until 13.00 on Monday of the following week and displayed by facility (Figure 1B).

### Data validation

The study investigators conducted data validation at all 87 study facilities in the first month and at 74 facilities in the last month of the study. Thirteen facilities, all in Ijara district, were not visited in the last month because of security reasons. A data collection tool was designed to record “gold standard” counts of OP, TM, PT and AM information extracted from facility registers by the study investigators during the on-site visits and those reported by registered health workers to the *SMS for Life* web data-base. These counts were made for week one at the beginning of the project, week 13 at the midpoint and week 26 at the end of the project. The dates of the specified weeks (Monday to Sunday) were determined and the appropriate outpatient department (for OP and AM counts) and laboratory and RDT registers (for TM and PT counts) obtained. The counts for each parameter were sequentially made from the relevant registers and recorded on the data collection form. Similarly, the dates of health worker *SMS for Life* database reporting for the specified weeks were established and reported counts for each parameter recorded. Discrepancies between the two counts were established by subtracting the health worker reported counts from the investigators’ gold standard count for the same week and the difference calculated as a percentage of the gold standard count. If the percentage discrepancy of any parameter was more than 10%, the health worker who reported the count was interviewed on possible reasons for the discrepancy.

### Data management and analysis

The analysis strategy was based on systems data stored in the *SMS for Life* web database and gold standard data collected for three study weeks by the investigators during the health facility data validation visits. SMS reported surveillance data for the 26-week intervention period were extracted from the *SMS for Life* web database and exported to Excel and STATA, version 11 (Stata Corp, College Station, TX, USA) for analysis. Data collected during the facility visits were entered in ACCESS and exported to STATA and Excel for analysis. Mean response rate was calculated as the proportion of correctly formatted SMS text messages sent by health workers to the web database each week and for the 26 weeks. Similarly, mean error rates were calculated as the number of wrongly formatted text messages sent each week and for 26 weeks. Accuracy of health worker SMS reported data was assessed by calculating the percentage discrepancy between the gold standard study investigators’ counts and the health worker reported counts for weeks 1, 13 and 26. To account for possible reading errors due to illegibility of hand writing in health facility registers, a cut-off point of ± 10% around the gold standard count was used to classify reports as accurate. To monitor malaria case management practices, three indicators: testing rate, test positivity rate and appropriate treatment ratio, were generated from the four data elements reported. Testing rates were calculated as the proportion of patients tested for malaria among all outpatients. Ideally, testing rate should be calculated as a proportion of suspected malaria cases. However, a crude testing rate calculated as a proportion of total outpatients was adopted due to the difficulty of obtaining counts of suspected malaria cases from routine data available at the time of the study. Test positivity rates were calculated as the proportion of positive cases among tested patients while appropriate treatment was expressed as a ratio of the number of patients treated with anti-malarials to the number of test positives. Discrepancies between test positives and anti-malarial treatment are an indication of deficient malaria case management practices. Analyses were done overall for all districts and for each district individually.

### Ethical approval

The study was approved by the Kenya Medical Research Institute Ethical Review Committee; reference number SSC 2055. Verbal consent to use health worker personal phones was obtained from all registered health workers during the training while written informed consent to access facility records and conduct interviews was obtained from the same health workers during the facility visits.

## Results

### Response rates, formatting error rates and accuracy of data

**Figure 2:**
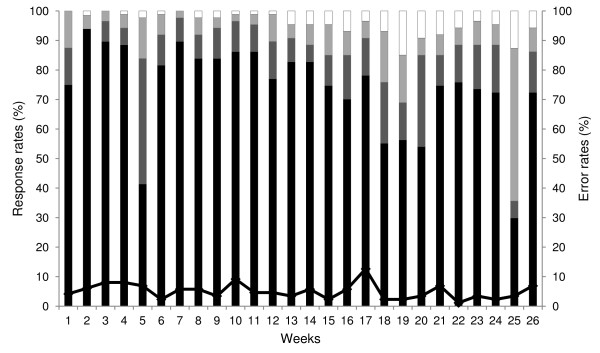
**Proportion of health facilities that responded to surveillance data request messages and SMS formatting errors by week.** Black bars show responses within 0–24 hrs; dark grey bars responses within 24–27 hrs (after reminder but within incentive period); light grey bars responses within 27 hrs-7 days (after the incentive period); white bars shows no responses, and black line shows SMS formatting errors.

Overall mean response rate across the five districts was 96%. Eighty-seven per cent of facilities responded within 27-hour incentive period, 9% responded after the incentive period while 4% did not respond at all (Figure 2). Delayed response rates were observed in week 5 and 25 due to network problems at the time, while those observed between week 18 and 20 coincided with end of year holidays when most health facilities were closed. Individual district mean response rates ranged from 93% in Machakos to 98% in Ijara with, respectively, 66% and 77% of facilities responding within the 27-hour incentive period. Msambweni and Vihiga districts had mean response rates of 97% and timely response rates of 76%. Manga district had a response rate of 94% and the highest timely response rate of 80%. Mean formatting error rates across all districts were low at 5% (range 3% in Msambweni to 6% in all other districts).

**Figure 3:**
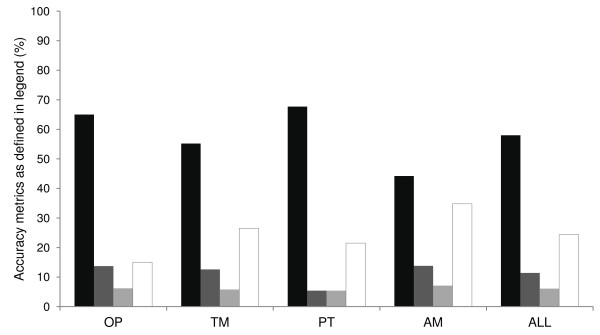
**Accuracy of SMS reported surveillance parameters.** Black bars show correctly reported surveillance reports (± 0-10%); dark grey bars show counts with discrepancy ± 10-20%; light grey bars show counts with discrepancy ± 20-30%, and white bars show counts with >30% discrepancy.

Accuracy of health worker SMS reported surveillance data was low with only 58% of parameters reported within the acceptable ± 10% discrepancy around the gold standard count. Indeed, nearly one quarter (24%) of parameters were reported with discrepancies of ≥30% (Figure 3). Within districts, accuracy of surveillance data reporting ranged from 53% in Msambweni to 60% in Machakos district. Manga and Vihiga accurately reported 56% and 57% of surveillance parameters, respectively. Accuracy of individual surveillance parameters ranged from 44% for anti-malarial treatments to 68% for malaria test positive reports while 55% of malaria tests and 65% of outpatient attendance counts were accurately reported. Reasons for inaccurate reporting for 98 out of 103 parameters were investigated during the last month of the project. Counting mistakes were the most common reason for inaccurate reports accounting for 54% of discrepancies. Other reasons included counting from un-recommended registers (17%), recording mistakes (8%), wrong determination of counting duration (6%), omitting some patient groups (4%), not conducting physical counts (4%), typing wrong number (4%), and including count of tests done in facilities not included in the study (3%).

### Testing rates, test positivity rates and appropriate treatment ratio

**Figure 4:**
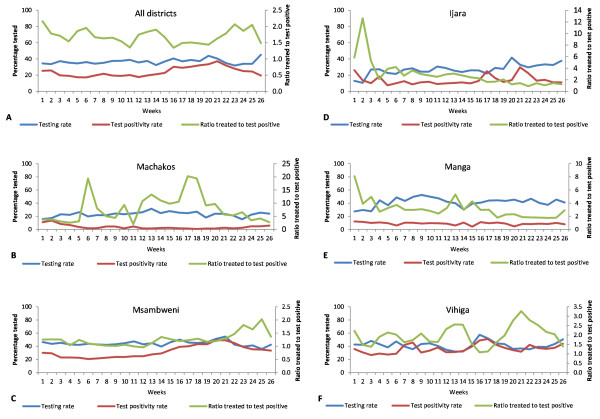
**Testing rates, test positivity rates and ratio of anti-malarial treatments to test positives overall and by district during the 26 weeks follow-up period. (A)** Overall testing rates, test positivity rates and ratio of anti-malarial treatments to test positives; **(B)** Testing rates, test positivity rates and ratio of anti-malarial treatments to test positives for Machakos Distict; **(C)** Testing rates, test positivity rates and ratio of anti-malarial treatments to test positives for Msambweni district; **(D)** Testing rates, test positivity rates and ratio of anti-malarial treatments to test positives for Ijara district; **(E)** Testing rates, test positivity rates and ratio of anti-malarial treatments to test positives for Manga district; **(F)** Testing rates, test positivity rates and ratio of anti-malarial treatments to test positives for Vihiga district.

A total of 320,642 outpatient visits were reported from 87 study facilities during the 26-week follow-up period. Of these, 117,892 (37%) were tested for malaria, 28,688 (24%) tested positive while 48,214 (15%) of all outpatients were treated with anti-malarials. Overall mean testing rate was 37% with minor weekly variations ranging from 32 to 45% (Figure 4A). Overall test positivity rate across five districts was 24% with a weekly range of 17-37%. A decrease in test positivity rate was observed in the first six weeks followed by an increase from week 15 to 21 and a declining trend towards the end of the project. Across all districts, mean ratio of anti-malarial treatments to test positives was 1.7:1 and varied greatly (weekly range from 1.3:1 to 2.2:1).

Within districts, mean testing rates were relatively low in Machakos (23%) and Ijara (27%) but higher in Vihiga (42%), Manga (42%) and Msambweni 44%. In Machakos district, an increase in testing rates was observed during the first five weeks of the project and thereafter a fluctuating pattern ranging from 16 to 32% (Figure 4B). Test positivity rate was high in the first two weeks but declined from the third week. Mean test positivity rate was 4% ranging weekly from 4 to 14%. Mean ratio of anti-malarial treatments to test positives was high at 7.9:1 with major weekly fluctuations ranging from 2.0:1 to 20.3:1. Msambweni district had relatively higher testing rates ranging from 36 to 55% with minor weekly fluctuations during the first 20 weeks and declining trend thereafter (Figure 4C). Mean test positivity rate was 32% ranging weekly from 21 to 49%. Test positivity rates declined gradually in the first six weeks but increased from the seventh week reaching a peak of 49% in week 20 and 21 and declined in the last weeks of the project. Mean ratio of anti-malarial treatments to test positives was 1.3:1 ranging weekly from 1.0 to 2.0. The ratio fluctuated around 1:1 concordance between week 6 and 13 but deteriorated from week 22 to 25 (1.5:1–2.0:1). In Ijara, testing rates ranged from 11 to 42%. Testing rates increased from the third week of the project and fluctuated throughout the follow-up period (Figure 4D). Mean test positivity rate was 14% ranging weekly from 8 to 30%. Mean ratio of anti-malarial treatments to test positives was 2.9:1 varying weekly from 0.9:1 to 12.6:1. The high ratio recorded in week 2 was possibly due to a reporting error. Ratios approaching 1:1 concordance were recorded from week 20 to the end of the project. In Manga, testing rate gradually increased from 27% in the first week to 52% in the ninth week followed by a declining trend through week 14 and a fluctuating pattern towards the end of the project (Figure 4E). Mean test positivity rate was 9% ranging weekly from 4 to 12%. Mean ratio of anti-malarial treatments to test positives was 3.1:1 with weekly variations ranging from 1.8:1 to 8.1:1. In Vihiga, testing rates ranged from 32 to 58% with declines observed between week 18 to 22 and increasing trends towards the end of the project (Figure 4F). The district recorded the highest mean test positivity rate of 36% with weekly variations ranging from 27 to 51%. Mean ratio of anti-malarial treatments to test positives was 2.0:1 reaching near 1:1 concordance in week 16 and 17.

## Discussion

This study found high and timely response rates of SMS reported surveillance data from peripheral health facilities across the five districts. However, accuracy of SMS reported surveillance data was low. Overall, 37% of outpatients presenting at the study facilities were tested for malaria with 24% of them testing positive. There was a high rate of overtreatment although short periods of near 1:1 concordance ratio between anti-malarial treatments and test positives were observed in three of the five study districts. These findings add to the growing evidence of the simplicity of using mobile phones to transmit real-time malaria surveillance data from peripheral health facilities to district and national level managers at higher response rates and shorter intervals than routine HMIS systems in Africa [7,9,11-13]. Use of SMS text message data requests to prompt health workers to send data may have contributed to the high response rate observed in the study. Timeliness in reporting may have been influenced by a 50 Kenya shillings airtime incentive awarded to health workers who responded to both stock [17] and surveillance data requests within the 27-hour incentive period although studies in Uganda and Madagascar have reported timely reporting without use of incentives [7,9]. Despite observed problems with mobile network connectivity (week 5 and 25) affecting timeliness of the reporting such events were rare over 26 weeks study period.

Accuracy of SMS reported surveillance data in the study was however suboptimal with only 58% of parameters accurately reported. Inaccurate data can compromise the benefits of mobile phone SMS-based reporting, potentially leading to significant misallocation of resources [18]. Further investigations of counting mistakes, which constituted 54% of the errors, revealed that 74% of wrong counts were done by untrained staff, mostly subordinate staff. *SMS for Life* project trained only one health worker per facility, who in most cases delegated the task of data extraction to untrained support staff. Health care providers in developing countries are usually overloaded with many tasks and disease reporting is not considered a high priority [19]. Challenges related to recording and extraction of data from multiple registers contributed to 25% of inaccuracy. For example, data on malaria testing were recorded in different registers depending on the type of test (RDT or microscopy) while data on outpatients were recorded in separate registers based on age of the patient. This complicated the data extraction process for *SMS for Life* leading to mistakes. Additional on the job training is urgently needed to increase the number of staff who can accurately report data. As shown previously, improved support supervision, regular data audits and continuous feedback to health facilities on data quality is also needed [20]. In addition, there is need to develop capacity of front-line health workers and their managers to interpret routine surveillance data and link it with appropriate response to correct any anomalies before transmitting them to higher levels of the health system [21].

Overall testing rates were low with some variations during the follow-up period without notable improvements over time. Although testing rates in this study were crudely measured using all outpatients as the denominator, low testing rates among malaria suspected patients have been commonly reported in other settings [22-25]. The apparent lack of improvement in testing rates even when diagnostics are available calls for an urgent need to explore reasons why testing is not sufficiently done and design interventions to address barriers to this important component of malaria case management.

The overall high ratio of anti-malarial treatments to test positives in this study is an indication of inappropriate case management practices that could be attributed to low testing rates and lack of corrective actions by district managers. A similar weekly mobile phone based surveillance system implemented in Zanzibar led to focused, aggressive data driven interventions prompting managers to conduct corrective actions involving outbreak investigations, rapid re-stocking of RDT and re-training of staff [26]. Such targeted interventions in response to surveillance data were however not observed in Kenya partly because of the relatively short implementation period of the project (26 weeks) as well as limitations of district managers to interpret and make decisions based on surveillance data. This lack of responsiveness by district managers could also be attributed to lack of predefined signals to prompt their action unlike the stock component of the study where red signals were displayed in the web database to highlight stock-out threats [17].

Analysis by district however, revealed short periods of improved case management practices with near 1:1 concordance ratio between anti-malarial treatments and test positives in Msambweni (weeks 6–13), Ijara (weeks 20–26) and Vihiga (weeks 16–17). Similar results were reported in Zambia where the ratio of anti-malarial treatment to confirmed cases varied over time but gradually stabilized to near 1:1 ratio as the duration of RDT use at health facilities increased [27]. Despite limitations of low accuracy of SMS reported in this study and the relatively short follow up period, the findings reveal important district specific trends in testing and use of anti-malarial drugs and show evidence of improved reporting rates with the use of mobile phones.

## Conclusions

This study has demonstrated the feasibility of using mobile phone based SMS text messages in Kenyan rural setting as a tool for improving timeliness and reporting of malaria surveillance data from facility level. However, the accuracy of SMS reported data was suboptimal, mainly due to staffing challenges and the complexity of extracting data from multiple registers. Future work should explore ways of improving the quality of data and providing feedback mechanisms to health workers.

## Competing interests

The authors declare that they have no competing interests.

## Authors’ contributions

SG collected and analyzed the data. SK, DM, AN, AW, ANW, and RZ contributed to the development of the intervention and implementation. SG, GJ, DZ, and RWS participated in study design, coordinated and helped in drafting the manuscript. All authors read and approved the final manuscript.
